# Editorial: Women in parasitology: 2021

**DOI:** 10.3389/fvets.2023.1153126

**Published:** 2023-03-28

**Authors:** Alessia Libera Gazzonis, Serena Cavallero, Simona Gabrielli

**Affiliations:** ^1^Department of Veterinary Medicine and Animal Sciences, Università degli Studi di Milano, Milan, Italy; ^2^Department of Public Health and Infectious Diseases, Sapienza University of Rome, Rome, Italy

**Keywords:** parasitology, women, gender equality, diversity, inclusion

Inclusion and gender balance in science have received increasing attention in recent years, as evidenced by the present “Women in Parasitology” Research Topic included in the Frontiers collection of articles “Women in Sciences.”

The participation of women in academia and scientific research remains underrepresented, with <30% of researchers being women worldwide (1). Women are even less represented in high-ranking academic positions, particularly in the Science, Technology, Engineering, and Mathematics (STEM) fields.

Various barriers, primarily of socio-cultural nature, are at the basis of the “leaky pipeline,” for which there is a disproportion in career advancement between men and women. Long-standing stereotypes historically discouraged women from entering the STEM world. Furthermore, there are still difficulties in reconciling an academic career with personal and family life, particularly during the highly demanding transition phase from early career to independent researcher, which frequently coincides with the will of motherhood. The COVID-19 pandemic has exacerbated this phenomenon, with the burden of family responsibilities increasing mostly for women; furthermore, the resulting economic losses have had a greater impact on women's employment, accounting for the largest number of job losses.

While more women are entering academia as junior researchers, only a small number hold senior positions. Similarly, in scientific publishing, women are underrepresented on editorial boards, accounting for 14% of editors and an even lower percentage of editors-in-chief (8%) (2). As a result of the lower representation of women in leadership positions, there are fewer female role models to inspire young female researchers.

Parasitology also aligns with other STEM-related fields, with women underrepresented among peer reviewers, editorial boards, and research councils, as recently reported (3). To promote the achievements of women parasitologists, Frontiers in Parasitology launched the Research Topic “*Women in Science: Parasitology*.” This Research Topic accepted eight original articles and one case report from a wide variety of research focuses and, to date, has over 10,000 total views and 1,258 article downloads. More than half of the 62 authors who participated in the Research Topic identified themselves as female. In particular, the participation of female researchers in their first career position should be emphasized, with the first position held by female authors in all included articles. Finally, there are four female researchers in senior positions, holding the last positions among the authors of the manuscripts. In the one-health perspective of parasitology, the current Research Topic includes articles focusing on parasites with great public health relevance and high zoonotic potential, or causative agents of important food-borne diseases (e.g., *Toxoplasma gondii, Taenia solium*, and *Echinococcus multilocularis*), as well as parasites affecting animal health and productivity. The main aspects were investigated with the aim of filling the gap in knowledge about complex host-parasite interactions and the actual limits of treatments and control strategies.

Rezende-Gondim et al. described an immunomagnetic separation (IMS) method for the purification of *T. gondii* and *Hammondia* spp. tissue cysts generated in cell culture. This method finds useful application in proteomic studies on *T. gondii* and other related parasites. Furthermore, given the large number of *in vitro* generated tissue cysts obtained, IMS also represents a promising alternative for *in vivo* generated cysts, thus helping to reduce the number of animals used for experimental purposes.

With similar potential outcomes, Li et al. carried out a comparative proteomic analysis of *T. solium* larval (cysticerci) and adult stages, providing reference values for studying the pathogenic mechanism of the two stages and the interaction with the host. Such studies are of great relevance, accounting for the ability of cysticerci to infect humans as well as adults.

With the aim of investigating novel alternative treatments against alveolar echinococcosis, Chaudhry et al. focused on the mitochondrial energy metabolism of *E. multilocularis*, among novel targetable pathways. The *in vitro* activity of 13 endochin-like quinolones against metacestodes and isolated germinal layer cells was screened. The results suggested a promising novel treatment approach for alveolar echinococcosis and other helminthiases, with implications for both human health and livestock production.

Meng et al. investigated the molecular mechanisms underlying the host-parasite interactions of a protein within the rhoptry kinase family of *Eimeria tenella*, one of the most pathogenic species among those causing avian coccidiosis. The transcriptomic analysis revealed that *E. tenella* rhoptry kinase family protein 17 (EtROP17) has several potential roles, including the modulation of parasite replication and contributing to defense against microbial infections.

Another important parasitic disease afflicting the poultry industry is avian trichomonosis caused by the flagellated protozoan *Trichomonas gallinae*. With the emergence of strains that are resistant to the standard treatment, alternative therapies for control are required. Bailén et al. studied the efficacy of several essential oils from Lamiaceae and Asteraceae plants against *T. gallinae*. The demonstration of good *in vitro* anti-trichomonal activity in the absence of cytotoxicity of the tested essential oils suggested their potential application in the control of this protozoan in avian production.

Nápravníková et al. addressed drug resistance with a study on the efficacy of the most commonly used drugs in the control of strongylid infections in horses, by means of fecal egg count reduction tests conducted on a large population of equines from the Czech Republic. While the macrocyclic lactones demonstrated excellent efficacy, the authors highlighted a resistance of strongylids against pyrantel embonate and fenbendazole, suggesting the urgency to review current practices used in parasitic control to slow or limit the spread of anthelmintic resistance.

In the field of companion animals, Györke et al. reported an intriguing clinical case of co-infection by *Notoedres cati* and *Aelurostrongylus abstrusus* in two domestic cats from Romania. The clinical symptomatology, the parasitological methodologies for the diagnosis of notoedric mange and aelurostrongylosis and the complementary laboratory tests, and the pharmacological treatment and management of the animals were described. The follow-up, with the complete resolution of symptoms in both subjects, confirmed the validity of the case management scheme.

Finally, studies by Zhang et al. and Hu et al. addressed aspects concerning the epidemiology of pathogens that cause important economic losses in the livestock sector worldwide. In the first study, the genetic diversity of *Enterocytozoon bieneusi*, a common opportunistic intestinal pathogen and cause of enteric disorders in immunosuppressed humans and animals, was investigated in Chinese pigs, with the identification of 15 known genotypes and the description of one novel genotype genetically closely related to the zoonotic genotype EbpB. The obtained genetic data on this Microsporidia, with both zootechnical relevance and zoonotic potential, provided the baseline data for the prevention and control of *E. bieneusi* in the pig industry. The seroepidemiological study by Hu et al. on *T. gondii* and *Neospora caninum* infections in goats from Southwestern China highlighted different patterns of distribution relating to geographical variables and factors regarding farm management. The findings suggested the need to implement tailored control measures to reduce the distribution of these protozoa, which cause reproductive failure and production losses in the caprine species worldwide.

The editors would like to thank the contributors and reviewers, who made it possible to produce a Research Topic of high scientific quality highlighting the impact of women researchers in the field of Parasitology and providing a space to disseminate science, especially in light of the International day of Women and Girls in Science (11 February). Here, young researchers driven by curiosity and highly scientific rigor have had the opportunity to promote their research activity; moreover, authors at different career levels represent role models for young female researchers. We, therefore, hope that this Research Topic has provided an opportunity to create new collaborations and networks of female researchers and to attract attention to the issues of gender equality, diversity, and inclusion in parasitology and in science in general.

## Author contributions

All authors listed have made a substantial, direct, and intellectual contribution to the work and approved it for publication.

## References

[1] UNESCO (2021). UNESCO Science Report: The Race Against Time for Smarter Development (Schneegans S, Straza T, Lewis J, editors). Paris: UNESCO.

[2] Liu F Holme P Chiesa M AlShebli B Rahwan T. Gender inequality and self-publication are common among academic editors. Nat Hum Behav. (2023). 10.1038/s41562-022-01498-1 36646836 PMC10038799

[3] Calvani NED De Marco Verissimo C Cantacessi C Clark E Kanduma E. Herminthology: promoting gender equity in science and parasitology. Trends Parasitol. (2023) 39:73–9. 10.1016/j.pt.2022.11.013 36526549

